# Comparative Transcriptome Analysis of *Galeruca daurica* Reveals Cold Tolerance Mechanisms

**DOI:** 10.3390/genes14122177

**Published:** 2023-12-04

**Authors:** Hongling Zhang, Feilong Sun, Wenbing Zhang, Xia Gao, Lei Du, Xiaopeng Yun, Yanyan Li, Ling Li, Baoping Pang, Yao Tan

**Affiliations:** 1 College of Horticulture and Plant Protection, Inner Mongolia Agricultural University, Hohhot 010019, China; zhanghongling@emails.imau.edu.cn (H.Z.); sunfeilong14747@163.com (F.S.); zhangwenbing@imau.edu.cn (W.Z.); liyanyan@imau.edu.cn (Y.L.); lling@imau.edu.cn (L.L.); pangbp@imau.edu.cn (B.P.); 2 Research Center for Grassland Entomology, Inner Mongolian Agricultural University, Hohhot 010019, China; 3 Key Laboratory of Grassland Resources, Ministry of Education, Hohhot 010010, China; skygxol@163.com; 4 College of Grassland, Resources and Environment, Inner Mongolia Agricultural University, Hohhot 010020, China; 5 Inner Mongolia Academy of Agricultural & Animal Husbandry Sciences, Hohhot 010031, China; poplarbreeze@163.com (L.D.); y8x7peng@163.com (X.Y.)

**Keywords:** *Galeruca daurica*, transcriptome, heat shock protein, glycolysis/gluconeogenesis, lipid synthesis, RNA interference

## Abstract

*Galeruca daurica* (Joannis) is a pest species with serious outbreaks in the Inner Mongolian grasslands in recent years, and its larvae and eggs are extremely cold-tolerant. To gain a deeper understanding of the molecular mechanism of its cold-tolerant stress response, we performed de novo transcriptome assembly of *G. daurica* via RNA-Seq and compared the differentially expressed genes (DEGs) of first- and second-instar larvae grown and developed indoors and outdoors, respectively. The results show that cold tolerance in *G. daurica* is associated with changes in gene expression mainly involved in the glycolysis/gluconeogenesis pathway, the fatty acid biosynthesis pathway and the production of heat shock proteins (HSPs). Compared with the control group (indoor), the genes associated with gluconeogenesis, fatty acid biosynthesis and HSP production were up-regulated in the larvae grown and developed outdoors. While the changes in these genes were related to the physiological metabolism and growth of insects, it was hypothesized that the proteins encoded by these genes play an important role in cold tolerance in insects. In addition, we also investigated the expression of genes related to the metabolic pathway of HSPs, and the results show that the HSP-related genes were significantly up-regulated in the larvae of *G. daurica* grown and developed outdoors compared with the indoor control group. Finally, we chose to induce significant expression differences in the Hsp70 gene (*Hsp70A1*, *Hsp70-2* and *Hsp70-3*) via RNAi to further illustrate the role of heat stress proteins in cold tolerance on *G. daurica* larvae. The results show that separate and mixed injections of *dsHSP70A1*, *dsHsp70-2* and *dsHsp70-3* significantly reduced expression levels of the target genes in *G. daurica* larvae. The super-cooling point (SCP) and the body fluid freezing point (FP) of the test larvae were determined after RNAi using the thermocouple method, and it was found that silencing the Hsp70 genes significantly increased the SCP and FP of *G. daurica* larvae, which validated the role of heat shock proteins in the cold resistance of *G. daurica* larvae. Our findings provide an important theoretical basis for further excavating the key genes and proteins in response to extremely cold environments and analyzing the molecular mechanism of cold adaptation in insects in harsh environments.

## 1. Introduction

Organisms living in cold regions are threatened by low temperatures in winter every year. For insects, chilling injuries result from complex physiological alterations such as loss of ions and water homeostasis, which participate in the disruption of neuromuscular functions, leading to chill coma [1]. Temperature is a key factor affecting the distribution and performance of insects [2], and low temperatures in winter are a threat to the occurrence and persistence of insect populations. Insects’ temperature adaptability is a key factor in determining their survival as well as their development, reproduction and spread and consequent crop loss in the following season [3]. Insects exposed to temperature stress may display alterations in physiology, morphology, behavior and life history [4]. Therefore, the survival strategies and adaptive mechanisms of insects in the harshest climates have been the focus of entomologists’ research [5]. 

Transcriptomics techniques (RNA-Seq) have been widely used to characterize gene expression changes in insects in response to a variety of environmental stresses, which is helpful not only to identify candidate genes for stress response but also to predict putative functions through the association of co-expressed or differentially expressed genes [6]. The rapid development of RNA-Seq technology has provided technical support for studying relevant gene expression in various species under adverse conditions [7]. Dunning et al. (2013) [8] used RNA-Seq to identify cold-responsive genes in the New Zealand alpine stick *Micrarchus hystriculeus* and then further compared the transcriptional profile expression levels of highland and lowland species populations to low temperatures, suggesting that cuticle modification may be associated with the colonization and development of cold-tolerant phenotypes in highland populations in low-temperature environments [9]. Liu et al. (2022) analyzed the transcriptome of *Calliptamus italicus* and found that hypothermia can induce the up-regulated expression of a large number of genes in eggs to alleviate hypothermia damage. Tusong et al. (2017) [10] analyzed the transcriptome of naturally adapted *Microdera punctipennis*, showing that most genes were up-regulated in eggs and indicating that the signaling and metabolic pathways associated with these genes play an important role in the overwintering process. Govaere et al. (2019) [11] reported that *Leptinotarsa decemlineata* can overcome unfavorable winter conditions by increasing keratin and HSP70 expression and adjusting transcription-related processes. Currently, RNA-Seq technology is widely used to study gene expression changes in response to low-temperature stress in multiple insect species, such as *Solenopsis japonica* [12], *Tamarixia radiata* [13], *Anoplophora glabripennis* [14], *Calliptamus italicus*, *Gomphocerus sibiricus* [15], *Liriomyza trifolii, Liriomyza sativae* [16], *Callosobruchus chinensis* [17], *Plutella xylostella* [18] and *Nysius groenlandicus* [19]. These studies have shown that temperature alterations can lead to changes in the expression levels of a range of genes involved in transcription, metabolism, development and especially heat shock proteins. 

The larvae of *Galeruca daurica* (Joannis), living in Mongolia, Russia (Siberia), Korea, Inner Mongolia, Xinjiang and Gansu Province in China [20,21], mainly feed on onion plants such as *Allium mongolicum*, *A. polyrhizum* and *A. ramosum* [22,23]. Since 2009, the extensive outbreaks of this pest have caused significant economic losses to rangelands and grasslands in Inner Mongolia [24]. *G. daurica* occurs in one generation each year and overwinters as eggs, and the larvae emerge as early as April, when the temperature fluctuates acutely and even the lowest temperature decreases below −10 °C in its natural habitats [25]. The overwintering eggs are strongly tolerant to extremely low temperatures, with a lower median lethal temperature (LT_50_) of −33.08 °C [26], and the larvae in spring are also highly tolerant to low temperatures, with an LT_50_ of −10.1 °C [27]. Rapid cold induction can significantly enhance the super-cooling capacity of *G. daurica* larvae [28]. Tan et al. (2017b, 2018) [29,30] reported that *GdHSP10*, *GdHSP60* and *GdHsp70* were associated with the growth and development of this insect and could be induced by both heat and cold stress; thus, these genes have important roles in the response of *G. daurica* to high- and low-temperature stress. Zhang et al. (2022) [31] showed that silencing *GdHSP60* and *GdHsp70* significantly reduced the cold tolerance of *G. daurica* larvae and also found that the expression levels of *GdHSP70-2* and *GdHSP70-3* varied at different developmental stages and in different tissues of *G. daurica*, therefore suggesting that different HSP70 genes play different roles at different developmental stages (Zhang et al., 2023) [32]. Based on the previous reports, it was hypothesized that the proteins encoded by these genes play an important role in the cold tolerance of insects. However, so far, few systemic studies have been conducted on the underlying molecular mechanisms of cold tolerance in *G. daurica*. 

To further explore the research related to cold tolerance and development, high-throughput RNA-seq technology was employed to conduct transcriptomic sequencing on larvae developed in different growth environments, and the obtained unigenes were analyzed with GO functional annotation, KOG annotation and the KEGG metabolic pathway. Specifically, we discovered that the differentially expressed genes were mainly enriched in the glycolysis/gluconeogenesis pathway, fatty acid biosynthesis pathway and the production of heat shock proteins (HSPs), which we proposed to be the possible main mechanism of *G. daurica* in responding to low temperatures. Finally, we applied RNA interference technology and determined the super-cooling point and body fluid freezing point of the *G. daurica* larval population after knocking down several key HSP genes. Exploring genes related to cold tolerance at the transcriptional levels also provides an important theoretical basis for further research on the biological function of related genes and the molecular mechanisms of low-temperature adaptation.

## 2. Materials and Methods

### 2.1. Collecting of G. daurica Larvae and Indoor and Outdoor Rearing

The *G. daurica* eggs, collected from the grassland of XiIingol Xianghuang Banner, Inner Mongolia (44°62′ N, 115°80′ E), were divided into an experimental group (outdoor incubation) and a control group (indoor incubation); they were fed from October 2020 to March 2021 until they developed into the first- and second-instar, respectively. The outdoor incubation was carried out on the farm of Inner Mongolia Agricultural University (40° 48′ N, 111°43′ E) with iron pot containers covered with soil and egg masses encased under the topsoil layer. The indoor incubation was carried out in a biomedical refrigerator at the Grassland Insect Research Laboratory of the Inner Mongolia Agricultural University (40°48′ N, 111°42′ E) at 4 °C. At the end of March 2021, the eggs of the two groups were transferred to an artificial climate chamber at 25 ± 1 °C, a relative humidity of 70 ± 5% and photoperiod of 14 L:10 D for incubation. They were moved indoors and outdoors again after hatching in 10 days, respectively. After hatching, the larvae were fed fresh *Allium mongolicum* leaves grown in a greenhouse; healthy and developmentally consistent first- and second-instar live insects incubated for 1 day were treated with liquid nitrogen and stored in a −80 °C refrigerator.

### 2.2. Indoor and Outdoor Temperature and Humidity Data Collection

The overwintering eggs of *G. daurica* were maintained in an artificial climate incubator (PRX-350C, Ningbo HaiShuSaiFu experimental instrument Co., Ltd., Ningbo, China) (25 ± 1 °C, RH = 70 ± 10%, L14:D10); the outdoor incubation was carried out in the farm of Inner Mongolia Agricultural University. The temperature and humidity data were collected and recorded using a Digital Data Track Intelligent Temperature and Humidity Recorder (179-TH, Shanghai Apresys Optoelectronics Co., Ltd., Shanghai, China), and the detailed recording information is shown in Table S1.

### 2.3. RNA Extraction, cDNA Library Construction and Transcriptome Sequencing

The total RNA was extracted from 20 mg of first- and second-instar larvae samples of *G. daurica*. Each treatment had three independent biological replicates, according to the instructions of the RNAiso Plus (9108Q, TaKaRa, Kyoto, Japan) kit, and the concentration and quality of total RNA were detected via an ultra-micro spectrophotometer and 1% agarose gel electrophoresis. The RNA was reverse-transcribed into cDNA using a PrimeScriptTM RT Reagent Kit with gDNA Eraser (6210A, TaKaRa, Kyoto, Japan) and stored at −20 °C for later use. The conserved samples were sent to Guangzhou Kidio Biotechnology Co. (Gene De novo Biotechnology Co., Ltd., Guangzhou, China). The transcriptome sequencing included first- and second-instar indoor-reared and outdoor-reared larvae, and three replicates were set for each treatment; a total of 12 larval cDNA libraries were constructed. After repairing the cDNA double ends with the addition of junctions, 200 bp cDNA sequences were screened with AMPure XP beads, PCR-amplified and purified, and then transcriptome sequencing was carried out using the Illumina NovaSeq 6000 high-throughput sequencing platform. After filtering the raw reads, checking the sequencing error rate and checking the GC content distribution, the clean reads were obtained and used for all downstream analyses. The clean reads were assembled using Trinity Release 2.13.1 software to obtain unigenes, N_50_ value and sequence length, and the Benchmarking Universal Single-Copy Orthologs (BUSCO) online platform (http://busco.ezlab.org/) (accessed on 10 March 2021) was utilized to evaluate transcriptome quality. The raw data were submitted to the NCBI Sequence Read Archive with accession number: PRJNA1017636.

### 2.4. Quantitative qRT-PCR for Verification

Quantitative real-time PCR (qRT-PCR) was performed to confirm DEGs in cDNA libraries constructed from indoor and outdoor first- and second-instar larvae, respectively. We selected 8 DEGs in the pathways related to the metabolism and heat shock proteins of *G. daurica* for qRT-PCR to verify the expression of these genes in *G. daurica* larvae. Quantitative PCR primers were designed with Primer-BLAST (https://www.ncbi.nlm.gov/tools/primer-blast/index.cgi) (accessed on 13 April 2023) (Table 1) and the succinate dehydrogenase (SDHA) gene was selected as the internal reference gene [33]. The qRT-PCR reaction system was performed according to the GoTaq^®^ qPCR Master Mix (A6001, Promega, Madison, WI, USA) instructions for configuration. A qRT-PCR instrument (FTC-3000, Funglyn Biotech, Toronto, ON, Canada) was used for the assay. The reaction program consisted of pre-denaturation at 95 °C for 10 min, denaturation at 95 °C for 15 s, annealing at 60 °C for 1 min and extension at 72 °C for 30 s for 40 cycles. A relative quantitative analysis was performed using the 2^−ΔΔCt^ method, and the experimental data were analyzed for the comparative significance of differences using Graph Prism 9.0 software.

### 2.5. dsRNA Synthesis

Based on the sequences of *HSP70A1* (Genebank Accession No: M76613.1), *HSP70-2* (Genebank Accession No: MZ853083) and *HSP70-3* (Genebank Accession No: OK585088) from the transcriptome database, specific primers were designed for PCR amplification (Table 1). The PCR reaction system (25 μL) consisted of 1 μL of cDNA template, 1 μL of each upstream and downstream primer (10 pmol/L), 12.5 μL of Premix TaqTM and 9.5 μL of RNase-Free ddH_2_O. The reaction program consisted of 94 °C for 3 min; 94 °C for 30 s; 58 °C for 60 s; 72 °C for 2 min, cycling 30 times; and 72 °C for 10 min. The test target genes verified via sequencing were entered into the online website http://www.flyrnai.org/cgi-bin/RNAi_find_primers.pl (accessed on 15 April 2023) and *dsRNA* primers containing the T7 promoter sequence at the 5′ end were designed (Table 1). The *dsRNA* was synthesized according to the instructions of the T7 Ribo MAXTM Express RNAi System and the concentration was measured, after which 1% agarose gel electrophoresis was used to detect whether the molecular weight size of the synthesized *dsRNA* was consistent with the expected one. Subsequently, the *dsRNA* was stored at −80 °C. Based on the results of Zhang et al. (2022) [31], when the concentration of *dsRNA* was 1000 ng/μL, the inference effect was best.

### 2.6. Microinjection of RNAi

The second-instar larvae with the same developmental stage (first day of molting) were selected, and soil and dust were brushed from their epidermis with a brush and temporarily fastened in clean slides with solid adhesive. The 0.4 μL (1000 ng/μL) *dsRNA* was injected into the 8th abdominal segment of the larvae using a microinjector (SHTMADZU, Kyoto, Japan), the larvae were placed into centrifuge tubes with air holes to be reared individually after injection, and their mortality was observed [33,34]. Two or more target genes interfering with the injected *dsRNA* were mixed in a 1:1 ratio and sampled at treatment 0, 24, 48, 72 and 96 h, with injections of sterilized water and *dsGFP* serving as blank and negative controls, respectively.

### 2.7. Relative Expression of HSP70A1, Hsp70-2 and Hsp70-3 after RNAi

Based on the sequencing of the *HSP70A1*, *Hsp70-2* and *Hsp70-3* cDNA, specific primers were designed for qPCR detection (Table 1). The qPCR reaction system (10 μL) consisted of 1 μL of cDNA template, 0.2 μL each of forward and reverse primers (10 μmol/L), 5 μL of SYBR Green Real-time PCR Master Mix Reaction Solution, and 3.6 μL of sterilized water. The internal reference genes were the same as in 2.6, and the primers are shown in Table 1. Three replicates were treated in each section of 2.6, with 20 individuals in each replicate. A q-PCR instrument (FTC-3000, Funglyn Biotech, Canada) was used for determination. The reaction program consisted of 95 °C pre-denaturation for 10 min; 95 °C denaturation for 15 s; 60 °C annealing for 1 min; and 72 °C extension for 30 s, 40 cycles.

### 2.8. Determination of Super-Cooling Point and Freezing Point

In order to test the silencing effects of *GdHsp70A1*, *GdHsp70-2* and *GdHsp70-3* on the cold tolerance of second-instar larvae of *G. daurica*, the thermocouple method was used to determine the super-cooling point (SCP) and freezing point (FP). A low-temperature incubator (LRH-100C, Shanghai Yiheng Instrument Co., Ltd., Shanghai, China) and multi-circuit automatic temperature recorder (TP9024U, Shenzhen Topray Electronics Co., Ltd., Shenzhen, China) were used. The test larvae were placed in a pipette gun tip stuffed with a small amount of degreased cotton, and the thermometer thermal probe was inserted so that the thermal probe could be fixed in the gun tip, precisely close to the larval body after 24 h of treatment in the abdominal segment, after which they were placed in a low-temperature incubator. We set a program to reduce the temperature in the incubator from room temperature to −40 °C at a rate of about 1 °C/min, during which the changes in body temperature were recorded [28,35]. Each treatment was replicated three times with 20 larvae per replication.

### 2.9. Gene Functional Annotation

To annotate the *G. daurica* transcriptome, we performed a BLAST search against the non-redundant (NR) database in NCBI, SWISS-PROT, KEGG and COG with an E-value cut-off of 10^−5^. We annotated the motifs and domains using InterPro [36]. Gene Ontology terms were assigned using Blast2GO [37] through a search of the NR database [38]. Several public protein databases were used, such as UniProt/Swiss-Prot [39], Cluster of Orthologous Groups of Proteins [40] and the Kyoto Encyclopedia of Genes and Genomes [41].

### 2.10. Normalization of Gene Expression Levels and Analysis of DEGs

Analyzing differential gene expression requires quantifying the expression levels of different expression genes (DEGs). Therefore, gene expression was normalized using reads per kilobase per million mapped exonic regions (FPKM) [42]. The FDR values were adjusted based on the method of Benjamini and Hochberg [43,44] after which the *p* values were derived. Genes were indicated as DEGs when the adjusted *p* value was <0.05 and |log2 (fold change)| ≥ 1.

### 2.11. Enrichment and Analysis of DEGs by GO and KEGG

The GO classification system was used to clarify the potential roles of all DEGs. The results are represented by three separate hierarchies, “Biological Process” (BP), “Cellular Component” (CC), and “Molecular Function” (MF). Pathway enrichment analysis was performed using the KEGG database to further assess significantly enriched metabolic or signaling pathways, and pathways with *p* value < 0.05 were defined as significantly enriched DGEs [45,46].

## 3. Results

### 3.1. Gene Annotation Results and Bioinformatics Analysis

There were four groups of tested *G. daurica* larvae, namely indoor first-instar larvae (GdIn1), outdoor first-instar larvae (GdOut1), indoor second-instar larvae (GdIn2) and outdoor second-instar larvae (GdOut2), reared in the lab at constant temperature and outdoors in variable natural temperature conditions, respectively. The indoor and outdoor rearing groups were regarded as the control and experimental groups, respectively. The samples were sequenced and filtered to obtain clean data (Table S2); the results show that the proportion of Q30 was more than 93% in all cases (the base quality value Q30 corresponds to a base recognition accuracy of 99.9%), and the proportion of unrecognized bases (N) was 0, indicating that the sequencing data were reliable.

The clean data reads were mixed and assembled, and 103,788 unigenes were obtained with an average length of 874 bp and an N50 length of 1611 (Table S3). Among them, there were a total of 30,588 with a length of 200–300 bp (accounting for about 29.30% of the total unigenes), a total of 28,169 with a length of 300–500 bp (25.99%), a total of 21,959 with a length of 500–1000 bp (20.03%), a total of 12,577 with lengths between 1000 and 2000 bp (13.20%) and 10,495 with lengths greater than 2000 bp (11.48%) (Figure S1).

The assembled unigene sequences of *G. daurica* were compared with the Nr, KEGG, SWISS-PROT and KOG databases, the predicted amino acid sequences of the unigenes were compared with the Pfam database, and, finally, 37,446 unigenes with annotations were obtained (Table S4). Among them, there were 37,020, 31,632, 16,994 and 15,419 unigenes annotated in the Nr, KEGG, SWISS-PROT and KOG databases, respectively, accounting for 98.86%, 84.47%, 45.38% and 41.18%, respectively. Through the annotating information from the Nr library, we obtained the similarity between unigene sequences of *G. daurica* larvae and homologous sequences of related genera, among which the most matched sequences were homologous sequences of *Diabrotica virgifera*, accounting for 27.2% with a total of 10,053 sequences and the homologous sequences of *Mus musculus*, *Anoplophora glabripera*, *L. decemlineata* and other matching species, with less than 3000 homologous sequences (Figure S2).

### 3.2. Analysis of the Expression of Biosynthesis-Related and HSP-Related Genes in G. daurica

**Figure 1:**
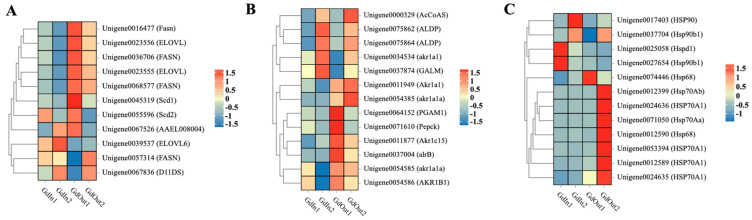
Heatmap of metabolism pathway in *G. daurica* larvae at different developmental periods indoors and outdoors. (**A**) Fatty acid biosynthesis pathway; (**B**) glycolysis/gluconeogenesis pathway; (**C**) heat shock protein pathway.

By analyzing the KEGG pathway enrichment of *G. daurica* larvae, we found that many pathways related to biosynthesis reactions were enriched, including lipid synthesis, glycometabolism and biosynthesis, amino acid metabolism, and so on. There are three main ways to protect insects from ambient cold temperatures: the fatty acid biosynthesis pathway, the glycolysis/gluconeogenesis pathway, and the HSP pathway. Among them, the glycolysis pathway stands out as glycogen is one of the major sources of energy for biological survival. Zhu et al. (2016) found that energy metabolism in insects increases with prolonged cold exposure; in particular, glycometabolism was sensitive to cold [47]. In 2017 year, they continued to report that carbohydrates in *Spodoptera litura* were rapidly depleted at low temperatures [48]. It is reported that long-chain fatty acid elongases (ELOs) play an important role in fatty acid metabolism in insects [49]. Duell et al. (2022) found that *Agrilus planipennis* can prevent internal ice formation by accumulating lipid content in the body, thereby increasing its own super-cooling point (SCP) [50]. Therefore, we selectively studied the pathways related to glycolysis/gluconeogenesis and fatty acid biosynthesis of *G. daurica*. After homogenizing the FPKM values of each unigene expression, the produced heatmaps show that the number of up-regulated genes was significantly more in the GdOut1 and GdOut2 groups than in the GdIn1 and GdIn2 treatment groups in the fatty acid biosynthesis pathway (Figure 1A). In the glycolysis/gluconeogenesis pathway (Figure 1B), some genes were highly expressed in GdIn1 and GdIn2 while expression in GdOut1 and GdOut2 was at low levels, but the number of up-regulated genes in GdOut1 and GdOut2 was still significantly more than that in GdIn1 and GdIn2, which is consistent with the lipid biosynthesis pathway. We also investigated the genes related to the HSP pathway; the heatmap shows (Figure 1C) that many HSP-related genes were significantly up-regulated in the GdOut2 treatment group compared with GdIn1, GdIn2 and GdOut1, such as *HSP68*, *HSP70A1*, *HSP70Aa* and *HSP70Ab*. Based on the heatmap results of gene expression, three *HSP70* genes were chosen for *RNAi* study.

### 3.3. Identification and Analysis of DEGs

**Figure 2:**
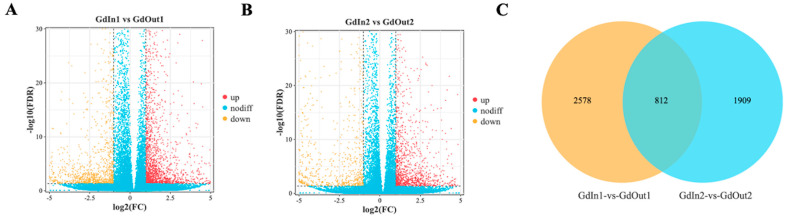
Volcano plot of DEGs between groups. (**A**) GdIn1 vs. GdOut1; (**B**) GdIn2 vs. GdOut2; (**C**) Venn diagram of shared DEGs between two comparing groups. Note: The horizontal ordinate represents the fold change of gene expression, and the vertical ordinate represents the statistical significance of the change. Each point represents a gene: the yellow points and red points represent differentially up-regulated and down-regulated genes, respectively, and the blue points represent a gene that does not show differential expression.

To identify DEGs in different treatment groups, we used DESeq2 software (1.20.0) to differentially analyze the read counts obtained in the gene expression level analysis. We defined GdIn1 as the first-instar control group and outdoor-feeding GdOut1 as the first-instar treatment group; meanwhile, GdIn2 was defined as the second-instar control group and outdoor-feeding GdOut2 was defined as the second-instar treatment group. In the GdIn1 vs. the GdOut1 group, there were a total of 3390 DEGs, of which 1958 were up-regulated for expression and 1432 were down-regulated for expression (Figure 2A). In the GdIn2 vs. the GdOut2 group, there were a total of 2721 DEGs, of which 1327 DEGs were up-regulated and 1394 DEGs were down-regulated for expression (Figure 2B). In addition, the identification of DEGs via Venn diagram (Figure 2C) revealed that 2578 genes were differentially expressed in the comparison of groups GdIn1 vs. GdOut1 and 1909 genes were differentially expressed in the comparison of groups GdIn2 vs. GdOut2, while there were a total of 812 DEGs in both groups.

### 3.4. GO Functional Enrichment of DEGs

**Figure 3:**
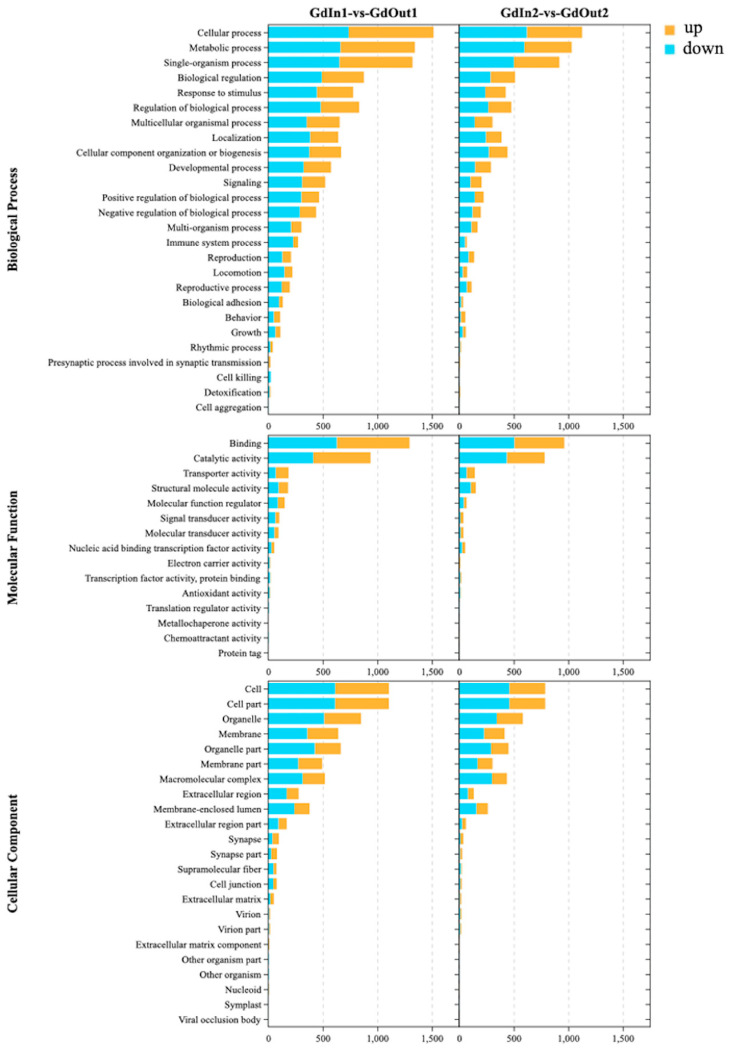
GO functional annotation of DEGs in larvae of *G. daurica* in different environmental conditions. DEGs of GO enrichment of the *G. daurica* transcriptome in groups. Note: tbhe *Y*-axis correlates to various gene functions, and the *X*-axis depicts the number of DEGs.

Compared with the indoor and outdoor first-instar (Figure 3), for biological processes, the top three GO terms with the highest enrichment were cellular process, metabolic process and single-organism process, with the number of differentially expressed genes (DEGs) being 1516, 1345 and 1323, respectively. In molecular function, the top two GO terms with the highest enrichment were binding and catalytic activity, with 1296 and 938 DEGs in the two pathways, respectively. In the cellular component, the top three GO terms with the highest enrichment were cell, cell part and organelle, with 1107, 1106 and 851 DEGs, respectively. In contrast, the indoor and outdoor second-instar comparison groups (Figure 3), whose enrichments were consistent with that of the first-instar comparison groups, had a lower number of DEGs in each pathway than that of the first-instar larval comparison groups. However, the number of DEGs in each pathway was less than that in the first-instar larval group, and the number of DEGs in the top 3 GO terms in the biological process were 1128, 1032 and 919, respectively. In terms of molecular function, the numbers of DEGs for binding and catalytic activity were 964 and 784, respectively. In the cellular component, there were 789, 789 and 584 genes differentially expressed in the cell, cell part and organelle, respectively.

### 3.5. KOG Annotation Analysis of DEGs

**Figure 4:**
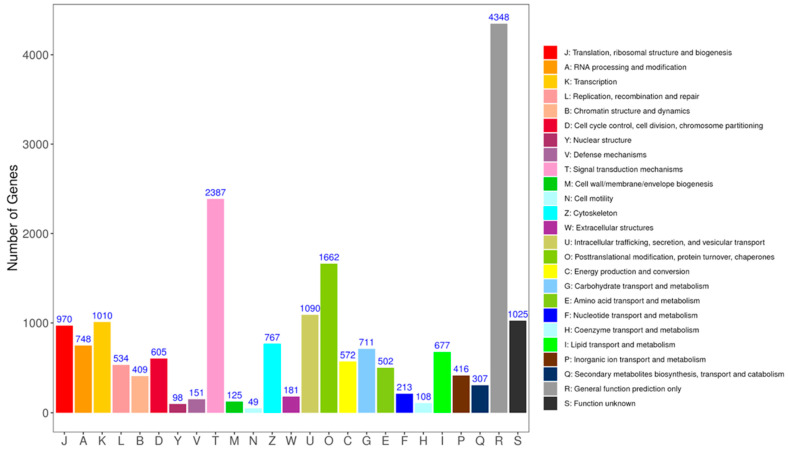
KOG annotation of DEGs in *G. daurica* larvae.

KOG homology classification of the *G. daurica* larval transcriptome unigenes yielded 19,665 gene annotations. In the larval taxonomic annotations, the categories with most gene annotations were general function prediction only in stage R and signal transduction mechanisms in stage T. The number of annotations were 4348 and 2387, respectively. Posttranslational modification, protein turnover and chaperones were the next largest categories in stage O, with 1662 annotations. There were also translation, ribosomal structure and biogenesis in stage J with 970 entries, and transcription in stage K with 1010 entries in the larval annotations (Figure 4).

### 3.6. The qPCR Validation of DEGs

**Figure 5:**
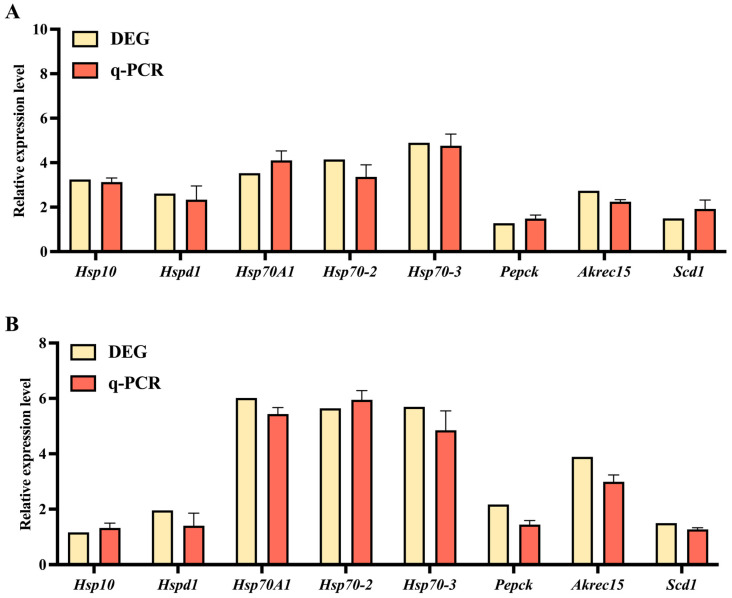
The qRT-PCR analysis of eight DEGs based on RNA-Seq analysis. (**A**) The DEGs in GdIn1 vs. GdOut1; (**B**) the DEGs in GdIn2 vs. GdOut12.

To confirm the reliability of the RNA-seq data, we performed qRT-PCR on eight DEGs selected from glycolysis/gluconeogenesis, lipid biosynthesis and HSP-associated pathways, which have the functions of regulating the metabolism, development, cold resistance and so on of *G. daurica*. These genes include heat shock proteins (HSPs), such as *Hspd1*, *Hsp10*, *HSP70A1*, *Hsp70-2* and *Hsp70-3*; they also include genes related to glycolysis/gluconeogenesis and lipid biosynthesis, such as alcohol dehydrogenase (*Akr1c15*), phosphoenolpyruvate carboxykinase (*Pepck*) and stearoyl-CoA desaturase (*Scd1*). The gene expression trends from the qRT-PCR results were consistent with the RNA-Seq deep sequencing data (Figure 5); therefore, the expression of these genes via qRT-PCR confirmed the direction of change detected via DGE analysis.

### 3.7. Expression Levels of Hsp70A1, Hsp70-2 and Hsp70-3 in Second-Instar Larvae of G. daurica after RNAi Using Microinjection Method

**Figure 6:**
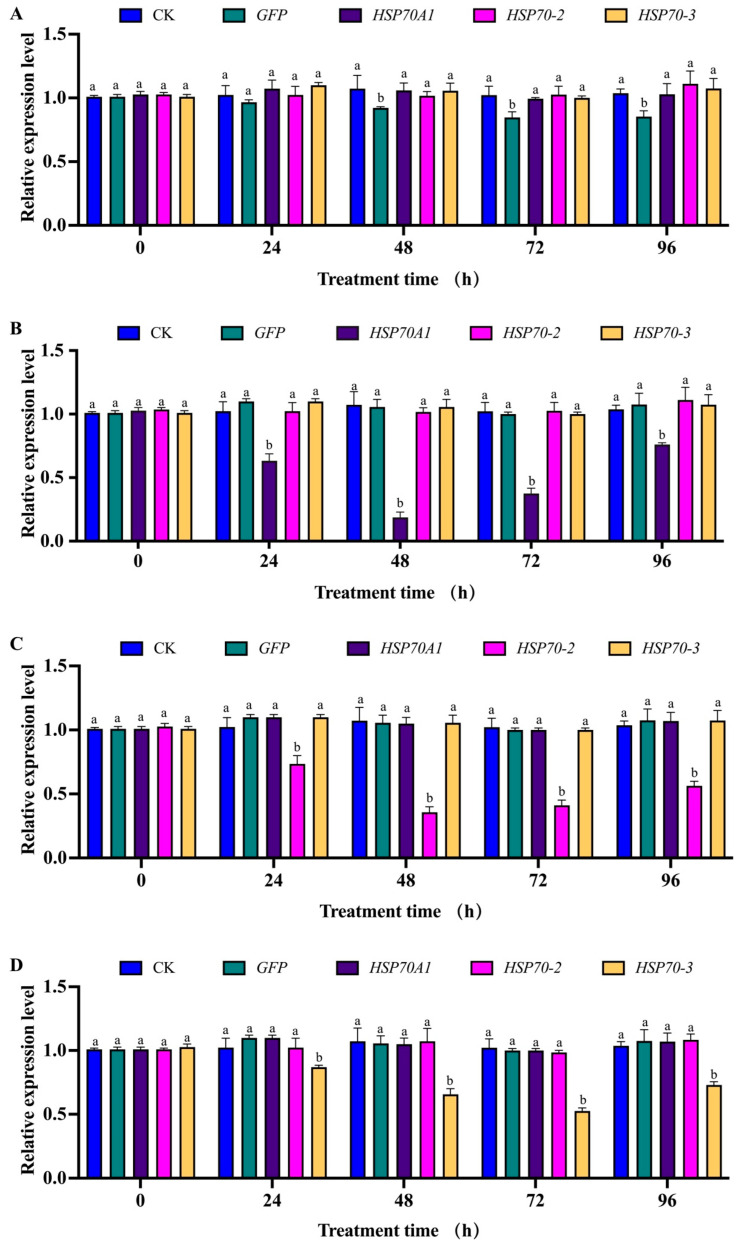

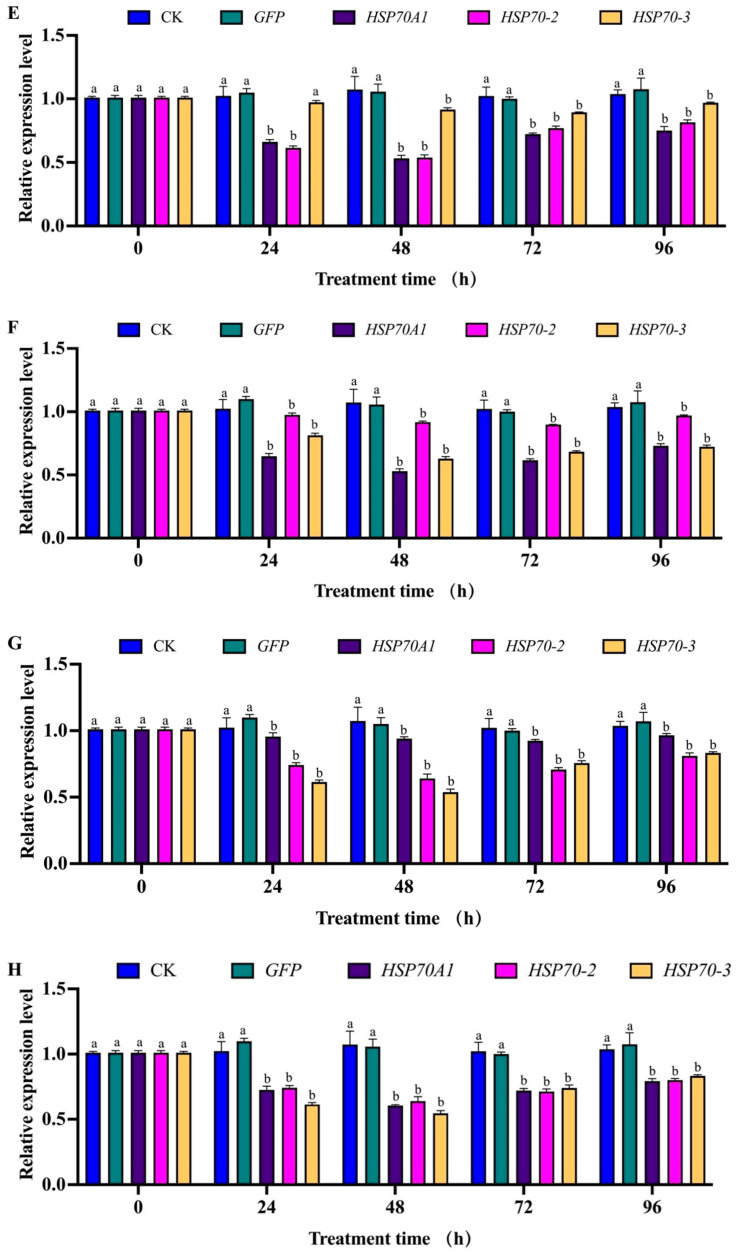
Relative expression levels of *Hsp70A1*, *Hsp70-2* and *Hsp70-3* in second-instar larvae of *G. daurica* after microinjection of *dsRNAs*. (**A**) Injection of *dsGFP*; (**B**) injection of *dsHsp70A1*; (**C**) injection of *dsHsp70-2*; (**D**) injection of *dsHsp70-3*; (**E**) injection of *dsHsp70A1* and *dsHsp70-2*; (**F**) injection of *dsHspA1* and *dsHsp-3*; (**G**) injection of *dsHsp70-2* and *dsHsp70-3*; (**H**) injection of *dsHsp70A1*, *dsHsp70-2* and *dsHsp70-3*. Note: Data in the figure are mean ± SE (*n* = 25). Letters above bars indicate significant differences among different treatments (*p* < 0.01) via ANOVA, LSD.

Compared with the *dsGFP* injection group (Figure 6A), the expression levels of *Hsp70A1*, *Hsp70-2* and *Hsp70-3* in the second-instar larvae of *G. daurica* significantly decreased after 24 h of injection (Figure 6B–D) and decreased to the lowest levels at 48 h and 72 h, with the expression levels decreased by 81.8% (*p* < 0.01), 65.3% (*p* < 0.01) and 48.7% (*p* < 0.01), respectively. The expression levels of these three genes still differed significantly after 96 h compared to the control (*p* < 0.01). We then mixed *dsRNAs* of two different genes for interference injection, such as *dsHsp70A1* and *dsHsp70-2*, *dsHspA1* and *dsHsp-3*, and *dsHsp70-2* and *dsHsp70-3* (Figure 6E–G, respectively). In the group injected with *dsHsp70A1* and *dsHsp70-2* (Figure 6E), the expression levels of the *Hsp70A1* and *Hsp70-2* genes decreased significantly at 24 h, 48 h, 72 h and 96 h (*p* < 0.01), and the expression levels of the *Hsp70-3* gene differed significantly at 48 h, 72 h and 96 h (*p* < 0.01). In the group injected with *dsHsp70A1* and *dsHsp70-3* (Figure 6F), the expression levels of *Hsp70A1*, *Hsp70-2* and *Hsp70-3* genes were significantly decreased within 96 h (*p* < 0.01). In the group injected with *dsHsp70-2* and *dsHsp70-3* (Figure 6G), the expression levels of *Hsp70A1*, *Hsp70-2* and *Hsp70-3* genes decreased significantly at four test intervals (*p* < 0.01). Finally, we injected the *dsRNAs* of three genes in a 1:1:1 mixing interference (Figure 6H), and the results show that the expression of *Hsp70A1*, *Hsp70-2* and *Hsp70-3* decreased to their lowest levels after 48 h of *dsRNAs* injection (*p* < 0.01), and the difference was still significant after 96 h (*p* < 0.01).

### 3.8. Super-Cooling Point and Body Fluid Freezing Point of G. daurica Larvae after RNAi

**Figure 7:**
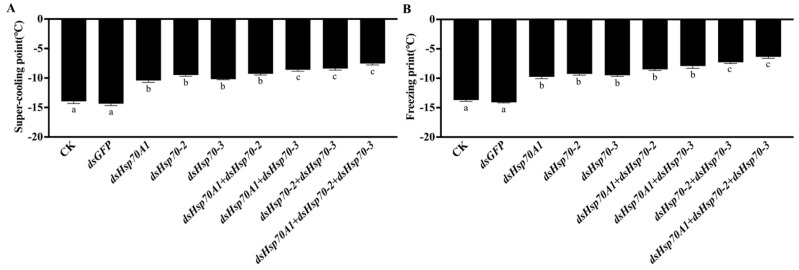
(**A**) The super-cooling point (SCP) and (**B**) freezing point (FP) of second-instar larvae of *G. daurica* after 24 h of RNAi of *GdHsp70A1*, *GdHsp70-2* and *GdHsp70-3.* Note: Data in the figure are mean ± SE (*n* = 20). Letters above bars indicate significant differences among different treatments (*p* < 0.01) via ANOVA, LSD.

The super-cooling point (SCP) and freezing point (FP) of *G. daurica* second-instar larvae were significantly elevated (*p* < 0.05) after 24 h following injection of *dsHsp70A1*, *dsHsp70-2* and *dsHsp70-3* (Figure 7). The SCP and FP of the second-instar larvae were −14.51 ± 0.21 °C and −14.07 ± 0.18 °C, respectively, after *dsGFP* injection, which was not significant (*p* > 0.05) compared with the control. The SCP of the second-instar larvae after injection of *dsHsp70A1*, *dsHsp70-2* and *dsHsp70-3* were −10.42 ± 0.41 °C, −9.53 ± 0.09 °C and −10.19 ± 0.45 °C, respectively. The FP of the second-instar larvae after *RNAi* of *Hsp70A1*, *Hsp70-2,* and *Hsp70-3* were −9.87 ± 0.12 °C, −9.21 ± 0.25 °C and −9.51 ± 0.31 °C, respectively, which were significantly higher compared with the control (*p* < 0.01). After injecting mixed *dsRNAs* of *Hsp70A1* and *Hsp70-2* genes, the SCP and FP of the second-instar larvae were −9.32 ± 0.53 °C and −8.51 ± 0.61 °C, respectively, which were significantly higher compared with the control (*p* < 0.01). After injecting mixed *dsRNAs* of *Hsp70A1* and *Hsp70-3* genes, the SCP and FP were −8.61 ± 0.71 °C and −7.86 ± 0.41 °C, respectively, which were significantly higher compared with the control (*p* < 0.01). After injecting mixed *dsRNAs* of *Hsp70-2* and *Hsp70-3* genes, the SCP and FP were −8.52 ± 0.72 °C and −7.35 ± 0.64 °C, respectively, which were significantly higher compared with the control (*p* < 0.01). After injecting mixed *dsRNAs* of three genes, *Hsp70A1*, *Hsp70-2,* and *Hsp70-3*, the SCP and FP were −7.52 ± 0.72 °C and −6.97 ± 0.84 °C, respectively, which were significantly higher compared with the control (*p* < 0.01) (Figure 7). In conclusion, the results show that separate and mixed injections of *dsHsp70A1*, *dsHsp70-2* and *dsHsp70-3* significantly reduced the super-cooling point and body fluid freezing point of second-instar *G. daurica* larvae.

## 4. Discussion

Temperature adaptation is an important factor in insect response to environmental change [51]; insects can adjust their own physiological and biochemical mechanisms by changing their cellular activity and metabolism to respond to changes in the external environment [52]. Cold stress is diverse, and different insect species have evolved different metabolic and physiological adaptations [53]. Maintaining cellular homeostasis, preserving cell viability and enhancing cellular tolerance by adjusting their metabolic status are the most important means of maintaining vital signs in insects at low temperatures [54]. Fatty acid biosynthesis plays a key role in the development of insects, not only in resisting environmental stresses in harsh environments but also in acting as an important antifreeze in low-temperature conditions [55], which is helpful in maintaining the stability of cell membranes, prevent cell freezing and rupture. This leads to the production and accumulation of fatty acids in insects at low temperatures and the development of cold tolerance [56]. On this basis, fatty acid biosynthesis is crucial for the survival of insects during the cold season [57]. A heat map related to lipid biosynthesis in *G. daurica* larvae (Figure 1B) shows that the fatty-acid-biosynthesis-related genes are expressed differently for indoor-reared and naturally developed *G. daurica* larvae. When *G. daurica* is alive in a thermostable environment, it synthesizes fewer fatty acids for growth and development because it does not have to cope with severe environmental stresses. On the contrary, when faced with environmental stresses, *G. daurica* larvae increase the intensity of lipid biosynthesis to improve their resistance to stresses.

The glycolysis/gluconeogenesis pathway plays a key role in the physiological processes of insects, especially in growth and development, flight energy supply, insect molting, metamorphosis and response to external environmental stresses. Through the heat map of glycolysis/gluconeogenesis in *G. daurica* larvae (Figure 1A), it can be concluded that certain genes may be highly expressed in an adverse environment to increase glycogen biosynthase and energy production to support the normal growth and development of *G. daurica* larvae. Regarding external environmental stress, other genes can also be induced to adjust the metabolism of larvae and reduce the energy consumption required for metabolic activities. Genes related to glycolysis/glycogenesis in *G. daurica* larvae perform different functions in different environments. This pattern of gene regulation provides *G. daurica* larvae with a strong adaption ability to the environment.

Insects surviving in cold environments exhibit cold tolerance due to the induction of heat shock proteins (HSPs) [58]. Cold stress can also cause denatured or misfolded proteins, leading to harmful aggregates and impaired function [59]. For *G. daurica*, the conformation and activity of their proteins and enzymes are easily damaged in severe and low-temperature environments on the grasslands, which causes rapid production of reactive oxygen species (ROS) in organisms [60]. HSPs play a key role in repairing and stabilizing these physiologically-active molecules [61]. When exposed to low temperatures, the expression levels of HSPs are up-regulated accordingly in response to physiological damage caused by cold [62]. The heat map reveals (Figure 1C) that these HSP-related genes have different expression patterns during the development of *G. daurica*. For example, the *HSP90* gene (Unigene0017403) was expressed at a higher level in second-instar larvae reared indoors, while it was down-regulated in second-instar larvae reared outdoors. The *HSP90b1* gene (Unigene0037704) tended to be up-regulated in second-instar larvae reared indoors and outdoors. In comparison, the *HSP70* gene showed high expression levels in second-instar larvae reared outdoors and low or no expression in larvae under other rearing conditions, which means that different types of HSPs have different expression patterns in insects, which is similar with the conclusion of Yang et al. (2021) [63], Zhang et al. [64] and Ruan et al. (2022) [65]. The results show that HSPs were sensitive to temperature in the growth and development process of *G. daurica.* HSPs also can respond to environmental changes promptly to maintain normal physiological state in insects [66]. This suggests that different types of HSP genes may have different roles and regulatory mechanisms in cold tolerance in insects [67]. The expression levels of HSP-related genes in *G. daurica* were significantly increased in cold environmental conditions and decreased in warm environments. These changes in HSP gene expression patterns not only reflect the perception intensity of *G. daurica* to low-temperature environments at different developmental stages but also reflect that it can adapt to low-temperature stress by regulating HSP expression [68].

The DEG volcano plots (Figure 2A,B) and Venn diagrams (Figure 2C) revealed that the number of DEGs in the first-instar larvae comparison group was significantly higher than in the second-instar larvae comparison group, which is because *G. daurica* first-instar larvae tend to occur in the colder early April [25]. During this period, although the temperature is gradually increasing, it is still relatively low. More genes are up-regulated to adapt to the lower environmental temperature for *G. daurica* larvae. These DEGs participate in various biological processes crucial for growth and development, contributing to the larvae’s ability to survive and thrive in cold environments. However, as the temperature continues to rise, *G. daurica* first-instar larvae gradually develop into second-instar larvae. At this stage, different genes are expressed to adapt to relatively mild late April. This is consistent with the results of GO functional enrichment analysis (Figure 3). In the KOG annotation analysis (Figure 4), it was found that a large number of genes in the *G. daurica* larvae were related to signal transduction, post-translational modification, protein turnover and molecular chaperones. This is consistent with the conclusion reported by Li et al. (2018) that heat shock proteins serve as highly conserved molecular chaperones, which can enhance the tolerance of organisms to stress [69]. However, the expression patterns and functional annotations of these DEGs can be influenced by various factors. Therefore, future studies need to combine new technological approaches to deeply investigate the influence of different ecological environment factors (such as climate, photoperiod, food source, etc.) on gene expression.

For insects living in low-temperature environments, the transient expression and regulation of HSPs are critical for maintaining their physiological functions and adaptive capacity [70]. The induced expression of HSPs in insects under stress is a natural defense mechanism against adverse environments, ensuring that insects can survive and develop better in cold environments [71]. Although many studies have reported that HSPs play a key role in the environmental adaptation of insects, only a limited number have employed RNAi to explore the impact of target genes associated with insect HSPs on insect cold tolerance. We used microinjection to introduce targeted *dsRNAs* into second-instar larvae, and the results show that RNAi can significantly reduce the expression level of HSP genes in insects. In addition, we determined the super-cooling point and freezing point of the larvae after silencing the target genes, and it was found that HSP gene interference can significantly reduce the cold tolerance of insects. This research result further confirms the important role of HSPs in insect environmental adaptation and reveals the potential application value of RNAi in studying the physiological and biochemical fields of insects. These findings provide new ideas and approaches for pest control.

## 5. Conclusions

In this paper, de novo transcriptome assembly of *G. daurica* by RNA-Seq were performed and the DEGs of first- and second-instar larvae grown and developed indoors and outdoors, respectively, were compared. The results show that the genes associated with glyconeogenesis, fatty acid biosynthesis and HSP production were up-regulated in the larvae grown and developed outdoors. In addition, significant expression differences in the Hsp70 gene (*Hsp70A1*, *Hsp70-2*, *Hsp70-3*) were conducted for RNAi to further illustrate the role of heat stress proteins in cold tolerance on *G. daurica* larvae. The separate and mixed injections of *dsHSP70A1*, *dsHsp70-2* and *dsHsp70-3* significantly reduced expression levels of the target genes in *G. daurica* larvae, and the super-cooling point (SCP) and the body fluid freezing point (FP) of the test larvae significantly increased, which validated the role of heat shock proteins in the cold resistance of *G. daurica* larvae.

## Figures and Tables

**Table 1:** List of primers used in this study.

Gene ID	Gene Name	Primer Sequences	Product Size	Purpose
NM_010477.4	*Hspd1*	F: CGCTCTGCTTCGGTGCAT	275	q-PCR
		R: CAGCAGCATCCAGTAAGGCA		
KY460464.1	*Hsp10*	F: GGCAACTACGAAAACGAAAGG	126	
		R: CCGCATTGTCTTCGAGGTTT		
KY460462.1	*Hsp70A1*	F: ACACTGGCCGTTTAAGGTGG	276	
		R: GCTGCTGCTGTTGGTTCATT		
MZ853083.1	*Hsp70-2*	F: GGAACCATTGCTGGTCTTGT	214	
		R: CGAAATCTTCACCTCCCAAA		
KO585088	*Hsp70-3*	TTCAACACTGGCCATTCAAA	218	
		TTTTGTAGCTTGTCTCTGTG		
NM_001109900.1	*Akr1c15*	F: GGAAGTAGATGCAGGTCGAGT	259	
		R: AACACCTGGCAGCTTGTGAT		
AY776138.1	*Pepck*	F: CGGTTTCTTCGGTGTTGCTC	243	
		R: TCCGCAGGAGTGCAAAATCT		
ON054187.1	*Scd1*	F: ATGCTGATCCTCACAACGCT	212	
		R: TAAATACATTGGGGCGGCGA		
KU240575.1	SDHA	F: GGGAGACCACAATCTCCTCA	192	
		R: AGCTGGTGCTCCTAAGTCCA		
	*dsHsp70-1*	F: taatacgactcactatagggGGTTCGACTCGTATTCCCAA	435	RNAi
		R: taatacgactcactatagggCCTTGGTCATTGGACGTTCT		
	*dsHsp70-2*	F: taatacgactcactatagggGGTTCGACTCGTATTCCCAA	334	
		R: taatacgactcactatagggCCTTGGTCATTGGACGTTCT		
	*dsHsp70-3*	F: taatacgactcactatagggAAGGAAGAGGATGAACGCCAGA	323	
		R: taatacgactcactatagggAATCCACCTGCTTGTTGACC		
	*dsGFP*	F: taatacgactcactatagggGGAGAAGAACTTTTCACTGG	560	
		R: taatacgactcactatagggAGTTGAACGGATCCATCTTC		

Note: lowercase letters stand for T7 promoter sequence.

## Data Availability

Data are contained within the article or Supplementary Materials.

## Supplementary Materials

The following supporting information can be downloaded at: https://www.mdpi.com/article/10.3390/genes14122177/s1, Figure S1: Unigene length distribution of *G. daurica* larvae; Figure S2: Annotation information of transcriptome data of *G. daurica* larvae and other species in Nr database; Table S1: The outdoor temperature and humidity data collection; Table S2: Summary of the RNA-Seq data from transcriptomes of first- and second-instar *G. daurica* larvae; Table S3: Transcriptome assembly results of *G. daurica* larvae; Table S4: Functional annotation of the unigene transcriptome of *G. daurica* larvae.
