# Supplementary figures and images for: Comparative Transcriptome Analysis of Galeruca daurica Reveals Cold Tolerance Mechanisms
Source: Genes (Basel). 2023 Dec 4;14(12):2177. doi: 10.3390/genes14122177 (PMC10742598; doi:10.3390/genes14122177)

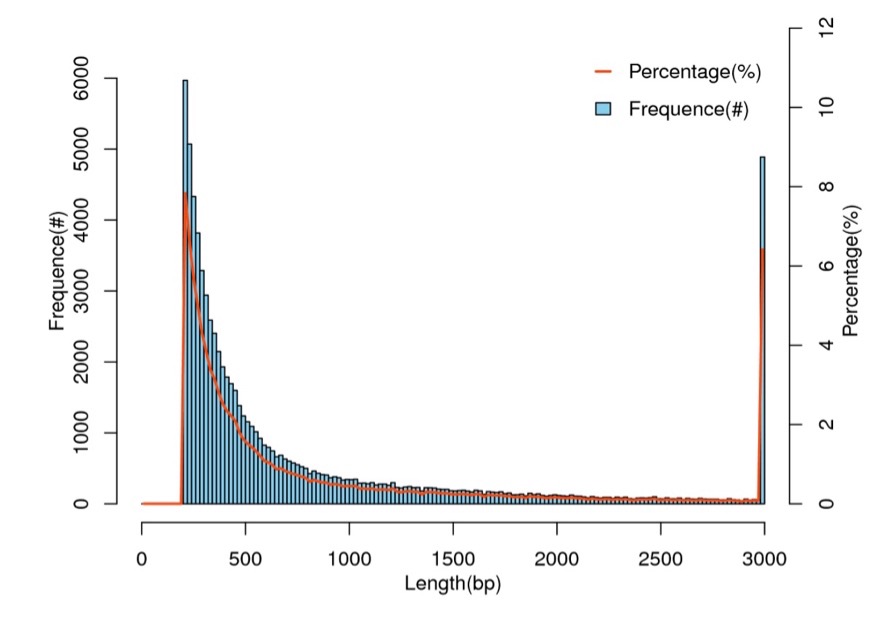

Supplement: Supplementary file 1 [file genes-14-02177-s001.zip › Figure.s1-Unigene length distribution of G daurica larvae.jpg]
